# Subchronic Exposure to Cadmium Causes Persistent Changes in the Reproductive System in Female Wistar Rats

**DOI:** 10.1155/2019/6490820

**Published:** 2019-12-17

**Authors:** Marzenna Nasiadek, Marian Danilewicz, Michał Klimczak, Joanna Stragierowicz, Anna Kilanowicz

**Affiliations:** ^1^Department of Toxicology, Medical University of Lodz, Muszynskiego 1, 90-151 Lodz, Poland; ^2^Department of Pathology, Medical University of Lodz, Pomorska 251, 92-213 Lodz, Poland

## Abstract

Cadmium (Cd) is an environmental toxicant and endocrine disruptor in humans and animals, and recent studies have illustrated that the uterus is exceedingly sensitive to Cd toxicity. The aim of the study was to investigate the influence of subchronic (90 days) oral Cd exposure in daily doses of 0.09-4.5 mg/kg b.w. on the balance of sex hormones by estimating estradiol (E_2_) and progesterone (P) concentrations in the uterus and plasma in comparison with the effects of 17*β*-E_2_. Additionally, the uterine weight, histopathological changes in the uterus and ovaries, the regularity of the estrous cycle, Cd bioaccumulation in uterine tissue, and selected biochemical parameters of oxidative stress were determined. A long period of observation (three and six months following the administration period) was used to assess whether the existing effects are reversible. The lowest dose of Cd caused effects similar to 17*β*-E_2_: an increase of E_2_ concentration in the uterus, endometrial epithelium thickness, and disturbed estrous cycle with estrus phase prolongation. The obtained results suggest that Cd causes nonlinear response. Higher doses of Cd caused a significant decrease in E_2_ concentration in the uterus and plasma, estrous cycle disturbances, endometrium atrophy, and structural damage in the ovaries. This dose additionally induces lipid peroxidation in the uterine tissues. It is noteworthy that a prolonged time of observation after terminating the exposure showed persistent changes in the concentration of E_2_ in uterine tissue, as well as alterations in estrous cycle phases, and an increase in lipid peroxidation in the uterus. Moreover, significant positive correlations between the plasma E_2_ concentration and endometrial epithelium thickness in all studied groups were found. In summary, subchronic oral Cd exposure of female rats may result in impaired fertility processes.

## 1. Introduction

Cadmium (Cd) is an important industrial and environmental pollutant. In addition, it should be taken into account that Cd pollution is global; hence, this metal has been placed 7th on the list of substances that pose a potential threat to human health due to their known or suspected toxicity [1]. Cd is used for the plating of steel, as a plastic stabilizer, as an electrode material in nickel-cadmium batteries, and as a material in semiconductors. Mining, smelting, and industrial use have resulted in the increased bioaccessibility of Cd in the environment, and anthropogenic sources are the most significant threat to human health [2]. Although Cd is used in a variety of manufactured products, for most people in the general population the primary sources of exposure are food and smoking tobacco [2, 3]. Cigarette smoking contributes between two and four *μ*g of Cd per pack. The European Food Safety Authority (EFSA) has estimated the average daily dietary exposure of Cd to be between 20.3 and 74.2 *μ*g/day per 70 kg person [4]. Specific groups at risk of consuming higher dietary Cd than is recognized as safe are children and vegetarians [5].

Women are thought to be at greater risk of increased Cd accumulation as the concentrations of Cd in the blood, tissue, and urine are higher than in males due to lower concentrations of iron [6–9]. In humans, Cd accumulates not only in the kidney and liver, but also in the reproductive organs [10–12]. To date, many *in vivo* studies indicate the toxic effects of Cd on female reproductive organs, including the endocrine system, the effects which seem to depend not only on the dose but also on the route of Cd administration [13–24]. However, the mechanism of Cd reproductive toxicity has not yet been elucidated completely, and there are still controversies with regard to the estrogen-like effect of this metal. Cd is listed as a metalloestrogen because of its ability to bind to the cellular estrogen receptor (ER) and hence mimic the actions of estrogens [25]. Johnson et al. [26] reported that Cd exposure in ovariectomized rats produced uterine hyperplasia, increased growth of the mammary glands, and the induction of hormone-regulated genes. Ali et al. [27] suggest that Cd exposure induces a limited spectrum of estrogenic responses *in vivo* and that, in certain targets, the effects of Cd might not be mediated via classical ER signaling through estrogen response element-regulated genes. Because of disturbed steroid hormone secretion, it is also suggested that the direct effect of Cd on the ovaries or the indirect effect of Cd on the hypothalamus-pituitary-gonadal axis should not be excluded. It was proved in *in vivo* studies that Cd administration leads to histopathological alterations in the ovary (degeneration of the corpus luteum, damaged and less numerous oocytes, and degeneration of granulosa cells) and uterus (an increase in the luminal epithelial height and in the endometrial thickness) [24, 28–30]. In addition, subacute Cd administration led to Cd accumulation and the induction of oxidative stress in rat ovaries and uterus [29, 31]. The results of our previous work indicate that subacute (30-day) exposure to Cd leads to histopathological changes in the ovaries and in the uterus, as well as disturbances in the concentrations of circulating steroid hormones, suggesting an antiestrogenic effect, which was associated with abnormalities in the estrous cycle in female rats [24]. Despite that environmental exposure to Cd is lifelong, data on the subchronic or long-term effects of Cd on the uterus is still limited. Thus, the aim of the study was to investigate the subchronic effects of Cd on the female rat reproductive system—by estimating the balance of sex hormones based on estradiol (E_2_) and progesterone (P) concentrations in the uterus and plasma in comparison with the effects of 17*β*-E_2_. Additionally, the uterine weight, histopathological changes of the uterus and ovaries, estrous cyclicity, Cd bioaccumulation in uterine tissue, and selected biochemical parameters of oxidative stress were determined. In order to discover whether the existing effects are reversible, we used a long period of observation in the exposed females, i.e., three and six months after the subchronic Cd administration period, after which the same parameters were determined.

## 2. Material and Methods

### 2.1. Animal Selection, Care, and Drug Treatment

Adult female Wistar rats (12 weeks old) were kept in polypropylene cages at a controlled temperature of 22 ± 1°C and relative humidity of 50-60% with free access to tap water and a diet low in phytoestrogen content (Ssniff R/M-H). The rats were allowed to acclimate for two weeks, during which the regularity of the estrous cycles was confirmed.

**Figure 1:**
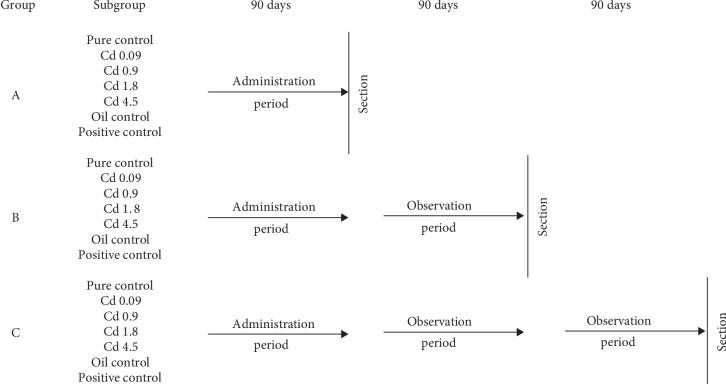
Experimental model.

The regularly cycling rats were separated into three experimental groups (each *n* = 56), A, B, and C, which were then divided into seven subgroups (each *n* = 8). The experimental design and dose regiment is summarized in Figure 1. The four Cd subgroups (from each of the following groups: A, B, and C) received Cd orally by gavage for 90 days (CdCl_2_, Sigma-Aldrich, St. Louis MO, USA) at different daily doses of 0.09, 0.9, 1.8, and 4.5 mg/kg b.w., which corresponded to 1/1000, 1/100, 1/50, and 1/20 LD_50_ (88 mg/kg b.w.) [32], respectively. The three control subgroups (from each of the following groups: A, B, and C) were administered distilled water (pure control), peanut oil (oil control), and 17*β*-E_2_ in a dose of 0.03 mg/kg b.w. (Sigma-Aldrich, St. Louis, MO, USA) dissolved in peanut oil (positive control). After 90 days of exposure, group A was sacrificed; however, groups B and C were subjected to three-month (90-day) and six-month (180-day) observation periods, respectively. The animals from the control subgroups received vehicle following the same protocol used for the rats exposed to Cd. The daily water intake and feeding habits of all the animals were carefully observed throughout the experimental schedule. During and following the treatment, all animals were observed carefully for mortality, body weight, and gross behavioral changes. All procedures conducted on the rats were approved by the Local Animal Ethical Committee of the Medical University of Lodz (LKE 46/LB/481/2009; 14/LB481/DLZ/2012).

#### 2.1.1. Euthanasia, Tissue Collection, and Preservation

The female rats which were in the estrus stage were weighed and underwent euthanasia after 90 days of exposure (group A), and after 90 days (group B) or 180 days (group C) of observation following a 90-day exposure. Blood samples were collected from all rats by heart puncture under light carbon dioxide anesthesia into Vacutainer tubes for metal analysis (S-Monovette, Sarstedt). The ovaries and the uterus were dissected out and weighed. For histological examination, part of the uterus and all of the ovaries were fixed in 10% formalin.

For Cd analysis, whole blood (1 mL) was kept in acid-washed cryotubes at -80°C, while the remaining part of the blood was centrifuged at 3000 × g (10 min, 4°C) to separate the plasma. Part of the uterus was stored at -80°C in cryotubes for Cd analysis. The plasma for hormone analysis and biochemical assays (sex hormones and total antioxidant status (TAS) concentrations) was stored at -20°C.

### 2.2. Cd Concentration Assessment

The whole blood and uterus Cd concentrations were measured using GFAAS (Hitachi Z-8270) with the Zeeman-type background correction, autosampler, and pyrocoated tube. Earlier, the whole blood and uterus samples were digested with ultrapure HNO_3_, using a microwave digestion system (MARSXpress, CEM Corporation, USA). To determine Cd concentrations, samples were prepared in duplicate. For each series of analyses, internal quality controls were used (analyses of reference samples: Seronorm Whole Blood Level 1 (Sero, Norway) and Bovine liver 1577b (National Institute of Standards and Technology)). The analytical quality control was in the range of reference values. The limit of detection (LOD) for Cd determined by GFAAS was 0.2 *μ*g/L or 0.2 ng/g wet tissue. Cd concentrations in whole blood and uterine tissue were expressed as *μ*g/L or *μ*g/g wet tissue.

### 2.3. Estrous Cycle Assessment

The estrous cycle was determined by cytological examination of vaginal smears obtained for two consecutive weeks prior to conducting the experiments and two weeks before section. The stages of rat estrous cycle were classified as proestrus, estrus, metaestrus, and diestrus according to the presence, absence or proportions of vaginal smears of three cell types: cornified cells (keratinized), epithelial cells, and leukocytes. The estrous cycle duration was calculated as the number of days between the estrus and proestrus stage [33, 34].

### 2.4. Histopathological Examinations

Part of the uterus and ovaries were fixed in 10% formalin for 24 h and then embedded in paraffin blocks, sliced into 5 *μ*m sections, and stained with hematoxylin-eosin (H&E) for the histopathological evaluation. The sections were examined under a light microscope (Olympus BX51; Olympus, Tokyo, Japan).

### 2.5. Measuring Endometrial Epithelium Thickness

The thickness of the epithelial layer was evaluated using a computer image analysis system consisting of a PC equipped with a Pentagram graphic tablet, an Indeo Fast card (frame grabber, true-color, real-time) produced by Indeo (Taiwan), and a color TV camera from Panasonic (Japan) coupled with a Carl Zeiss microscope (Germany). This system was programmed (MultiScan 18.03 software, produced by Computer Scanning Systems, Poland) to calculate the distance (semiautomatic function). In each case, measurements were performed in high-power monitor fields and then the mean endometrial epithelium thickness was calculated.

### 2.6. Biochemical Analysis

#### 2.6.1. Plasma Sex Hormone Concentration

The plasma E_2_ and P concentrations were determined by an electrochemiluminescence method using a Roche Diagnostic kit on a Cobas 2601 analyzer (LOD: E_2_ = 5 pg/mL; P = 0.03 ng/mL). The values reported are the sum of estradiol and estrone because chromatographic purification of the samples was not performed.

The concentrations of E_2_ and P in the rat tissue were determined using an ELISA kit according to the manufacturer's instructions, respectively: rat (E_2_) ELISA kit—catalog No. 201-11-0175, SRB (China), and (P) ELISA kit—catalog No. CSB-E07282r Cusabio Biotech Co., Ltd, (Japan). The sensitivities of the kits were E_2_ = 3.112 pg/mL and P = 0.25 ng/mL. The samples of uterus for hormone analysis were homogenized well to produce 10% homogenates in PBS and stored overnight at -20°C. For the assay of E_2_, the samples were centrifuged for 20 min at 2000‐3000 × g. For the assay of P, after two freeze-thaw cycles were performed to break the cell membranes, the homogenate was centrifuged for 5 min at 5000 × g at 2–8°C. The E_2_ and P assays in the supernatant were carried out immediately.

#### 2.6.2. Determination of Plasma TAS

The major antioxidant defences in plasma include ascorbate, protein thiols, bilirubin, urate, and *α*-tocopherol. Applying this method (TAS) allows to determine these major antioxidants in plasma. The plasma TAS was measured with a Ransel NX 2332 ready-made test (Randox Laboratories), according to the manufacturer's instructions. The TAS concentration was expressed as mM.

#### 2.6.3. Determination of Catalase (CAT) Activity in Uterus

The uterus tissues were rapidly excised and homogenized in an ice bath using phosphate-buffered saline (pH 7.4) with 0.01% digitonin using a Kika Labortechnik T-25 basic homogenizer. The homogenate was centrifuged at 10,000 × g for 30 min at 4°C. CAT activity in the supernatant of the uterus homogenate was measured using a CAT 240 colorimetric assay kit for CAT activity (Applied Bioanalytical Labs) according to the manufacturer's instructions. CAT activity was expressed as U/mg protein. Protein concentrations in supernatants were determined according to Lowry et al. [35].

#### 2.6.4. Determination of GSH in Uterus

The GSH concentration in the uterus (10% homogenate in phosphate-buffered saline pH 8.0) was determined according to Sedlak and Lindsay [36]. This method allows to assay nonprotein sulfhydryl compounds (NPSH), the sum of cellular glutathione and cysteine. Glutathione exists in thiol-reduced (GSH) and disulfide-oxidized (GSSG) forms. The GSH accounts for more than 90% of total NPSH [37], while the GSSG content is less than 1% of GSH [38]. The standard curve was obtained by using GSH, hence the results were expressed as GSH concentration (*μ*mol/g tissue).

#### 2.6.5. Determination of MDA in Uterus

MDA concentrations, an indicator of free radical generation, which increases at the end of the lipid peroxidation, were estimated according to Uchiyama and Mihara [39]. The concentration of MDA was expressed as nmol/g tissue.

### 2.7. Statistical Analysis

All biochemical data were analyzed by STATISTICA software (StatSoft, Poland). We used the Kruskal-Wallis one-way analysis of variance followed by a pair-wise comparison of selected means with the Mann-Whitney *U*-test. We used Spearman's rank correlation (*r*) to assess univariate associations.

In the estrous cycle, the statistical analysis was comprised of the following: (1) the mean length of the estrous cycle in the controls and the Cd-exposed females, and (2) the frequency of each of the four cycle phases. The one-way analysis of variance following Dunnet's test was used in the case of variance homogeneity, and the Kruskal-Wallis analysis of variance was followed by the nonparametric test in the case of heterogeneity. Frequency data were analyzed with the Fisher's exact probability test. The statistical significance was set at *p* ≤ 0.05.

## 3. Results

During the whole administration time (90 days) and observation periods (three or six months) no changes in animal behavior or appearance were noted; all rats survived until the termination of the study. There were also no significant changes in feed and water intake during the Cd administration or observation periods (data not shown). Moreover, integral toxicity parameters, such as body weight, selected organs' (liver, kidneys, and uterus) absolute and relative weights, and weight gain were unchanged in all groups (Supplementary [Supplementary-material supplementary-material-1] and [Supplementary-material supplementary-material-1]).

### 3.1. Cd Concentration

**Figure 2:**
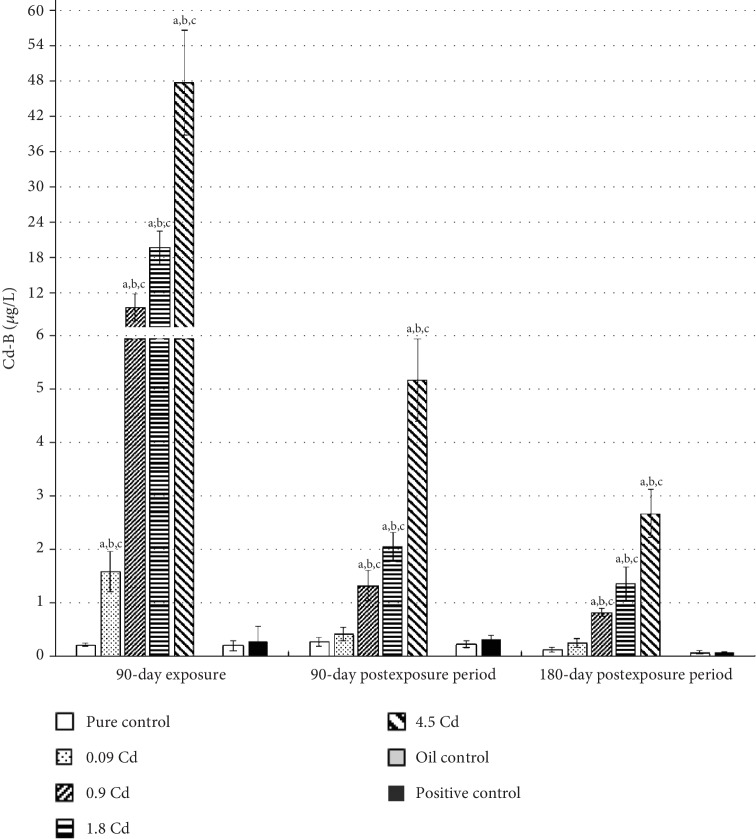
Cadmium concentrations in blood (Cd-B) following 90-day oral exposure to CdCl_2_ or 17*β*-estradiol (positive control), at the 90-day and 180-day postexposure periods. *p* ≤ 0.05 (a—vs. pure control group, b—vs. oil control, and c—vs. positive control).

Subchronic *per os* administration of Cd at the range of doses used in this study resulted in a significant dose-dependent increase in whole blood Cd concentration (Cd-B), as depicted in Figure 2. The mean Cd-B in rats from all pure control groups did not exceed 0.3 *μ*g/L. With the exception of the lowest Cd dose (0.09 mg/kg b.w.), significantly elevated Cd-B were maintained for both three and six months following the exposure. However, a decreasing trend in Cd-B could be observed in the postexposure period for all used doses (Figure 2). For the first three months of observation, the rate of Cd-B decrease was faster (about 10 times) than during the subsequent three months of observation (about twice). Only in the case of the lowest dose (0.09 mg/kg b.w.) did the Cd-B concentration decrease about three times after three months following the exposure period.

**Figure 3:**
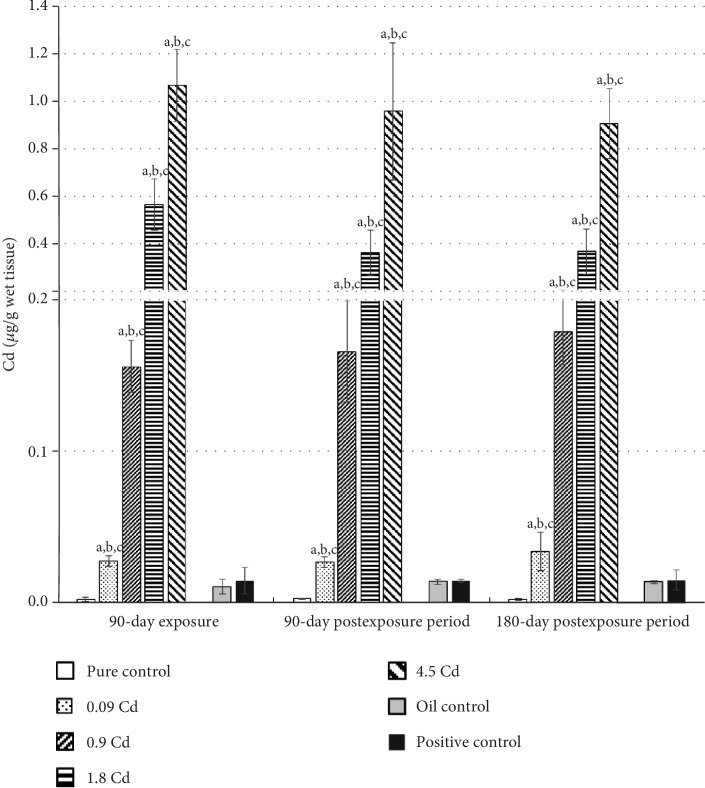
Cadmium concentrations in the uterus following 90-day oral exposure to CdCl_2_ or 17*β*-estradiol (positive control), at the 90-day and 180-day postexposure periods. *p* ≤ 0.05 (a—vs. pure control group, b—vs. oil control, and c—vs. positive control).

Like the whole blood, the concentration of Cd in the uterus was also dose-dependent (Figure 3). In the case of the uterus, the mean increase in Cd concentration, after 90 days' administration with Cd doses of 0.09, 0.9, 1.8, and 4.5 mg/kg b.w., was 15-, 80-, 320-, and 500-fold, respectively, compared to the control group. However, in contrast to Cd-B, all used doses caused a significant increase in Cd concentrations in this tissue, which was maintained at nearly the same concentration for up to six months after the end of the administration period. Moreover, in the examined females, a significant, strong correlation between the Cd-B and Cd concentrations in uterine tissues was observed after 90 days of exposure (*r* = 0.98; *p* ≤ 0.05) and after three (*r* = 0.92; *p* ≤ 0.05) and six months following termination of the exposure (*r* = 0.96; *p* ≤ 0.05).

### 3.2. Concentrations of Sex Hormones

**Figure 4:**
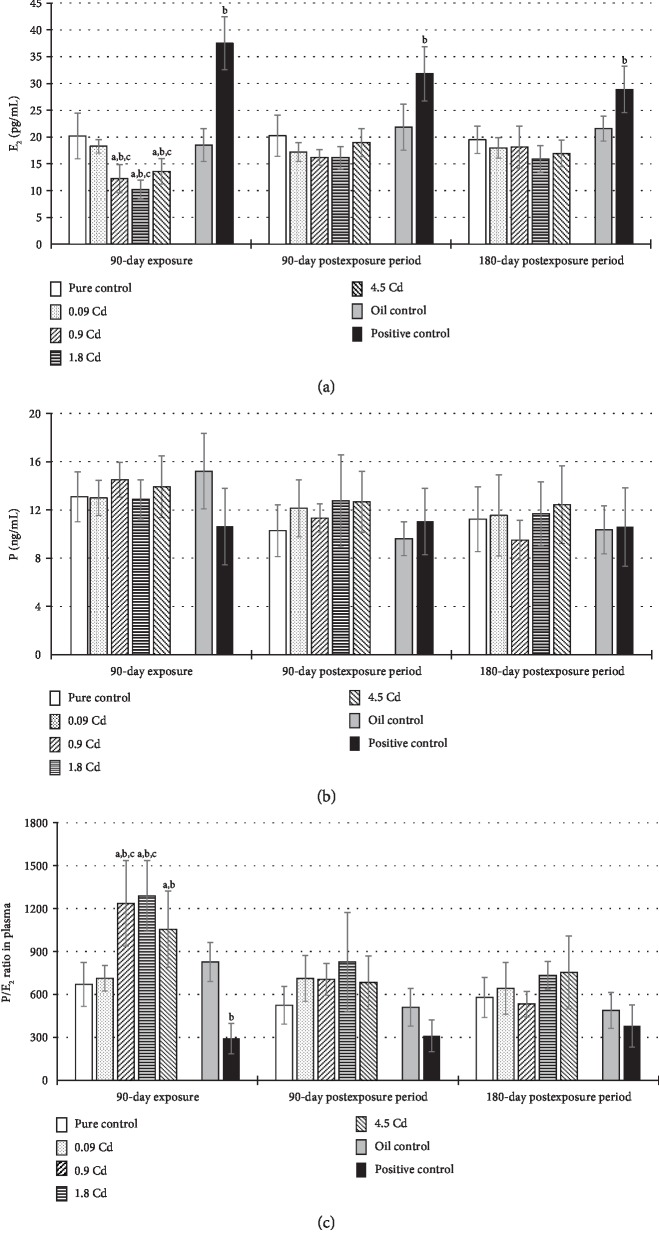
E_2_ (a) and P (b) concentrations and the ratio of P/E_2_ (c) in plasma following 90-day oral exposure to CdCl_2_ or 17*β*-estradiol (positive control), at the 90-day and 180-day postexposure periods. *p* ≤ 0.05 (a—vs. pure control group, b—vs. oil control, and c—vs. positive control).

**Figure 5:**
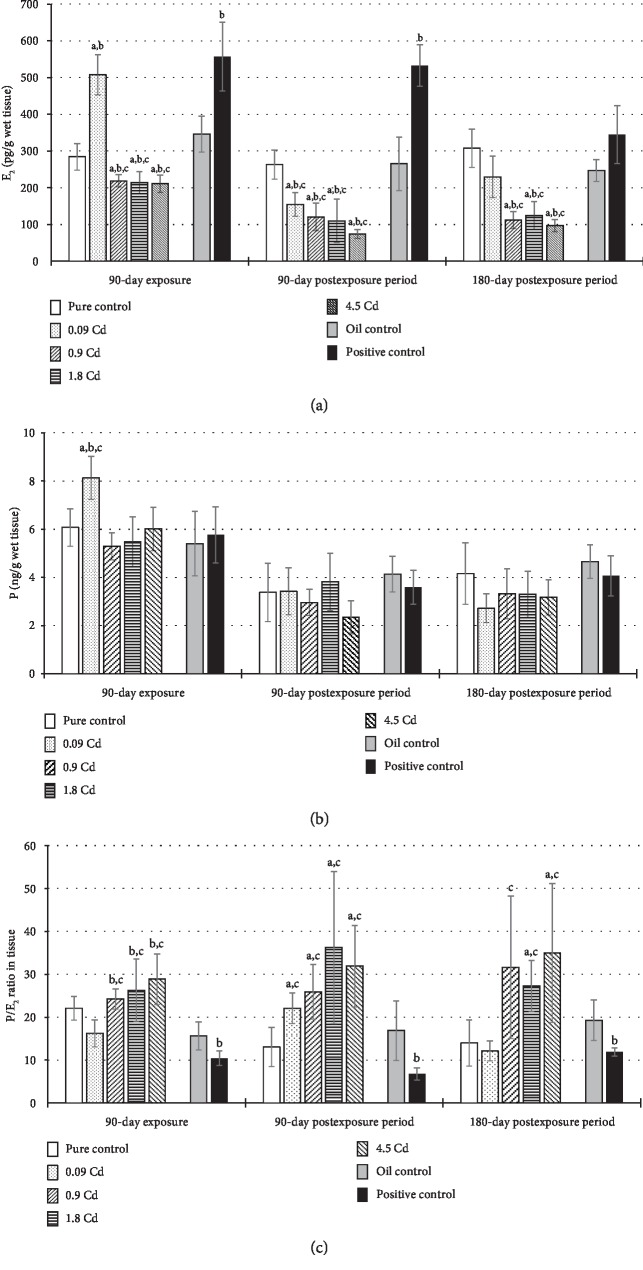
E_2_ (a) and P (b) concentrations and the ratio of P/E_2_ (c) in uterine tissue following 90-day oral exposure to CdCl_2_ or 17*β*-estradiol (positive control), at the 90-day and 180-day postexposure periods. *p* ≤ 0.05 (a—vs. pure control group, b—vs. oil control, and c—vs. positive control).

Concentrations of selected sex hormones (E_2_ and P) and their ratio (P/E_2_), both in the plasma and in the uterus tissue, are presented in Figures 4 and 5. The E_2_ plasma concentration significantly decreased after administration of Cd in doses 0.9-4.5 mg/kg b.w. (Figure 4(a)), but this effect was not observed in the postexposure periods. In the case of the uterus, only the lowest dose (0.09 mg/kg b.w.) and the positive control caused a significant E_2_ concentration increase in contrast to the other Cd doses, where the opposite trend was observed (Figure 5(a)). However, after 90 and 180 days of observation, in almost all animals exposed to Cd, a diminished concentration of E_2_ was noted (Figure 5(a)). In the positive control group, the remarkably higher concentration of E_2_ noted at the end of the exposure lasted up to six months in the plasma and up to three months in the uterus (Figures 4(a) and 5(a)).

The concentration of P in plasma seems to be unaffected by Cd administration (Figure 4(b)). In the uterus, only exposure to the lowest dose resulted in a significant increase in P concentration (Figure 5(b)). For a better illustration of the disturbed hormonal homeostasis, a P/E_2_ ratio was calculated (Figures 4(c) and 5(c)). As shown in the results from Figure 4(c), significant disturbances in the calculated ratio of hormones' concentrations (P/E_2_) in the plasma was observed only after 90 days of the exposure in the groups of rats administrated Cd at doses of 0.9–4.5 mg/kg b.w., which mainly result from a decrease of E_2_ concentration in the plasma of these females. However, in uterine tissue, significant changes were detected in the calculated ratio of P/E_2_ hormones in all assessed groups, which persisted until termination of the observation, with the exception of the group administered the lowest dose, in which the changes were not statistically significant (Figure 5(c)).

### 3.3. Estrous Cycle

The results of the estrous cycle analysis after exposure to Cd are presented in Table 1 and in Supplementary Figures [Supplementary-material supplementary-material-1]. For better readability of the observed disorders, which often occur in individual rats and sometimes there was no statistical significance in the group observed, in the figures, they are presented for each female rat individually. The highest number of both extended cycles and different phase lengths was recorded after 90 days of exposure in almost all groups administered Cd. The largest prolongation of the cycle length (5.6 ± 0.3 days) was noted in the positive control, which was caused by the prolongation of the estrus phase at the expense of the diestrus and proestrus phases. In the groups of female rats exposed to Cd in doses of 0.09-1.8 mg/kg b.w., a statistically significant prolongation of the cycle was also noted in 37.5-50% of the rats; however, it was conditioned not only by the estrus phase (doses of 0.09 and 1.8 mg/kg b.w.) but also by the diestrus phase (dose of 0.9 mg/kg b.w.). After the rats were administered the highest dose of Cd (4.5 mg/kg b.w.), no significant increase in the duration of the cycle length was observed, but the individual phases, especially diestrus and proestrus, were significantly extended and shortened, respectively.

After both three and six months of postexposure observation, a significant prolongation of the cycle length among the exposed groups was maintained only at the lowest Cd dose (0.09 mg/kg b.w.) among 25% and 37.5% of the rats, respectively. However, the statistically significant prolongation of the estrus phase was only observed after 180 days of the postexposure period (Supplementary [Supplementary-material supplementary-material-1]). Administration of a higher dose (0.9 mg/kg b.w.) both after 90 and 180 days of observation led only to a statistically significant extension of the estrus phase; however, it did not lead to significant changes in the duration of the whole cycle length (Supplementary Figures [Supplementary-material supplementary-material-1] and [Supplementary-material supplementary-material-1]). In the positive control, no changes were noted in the estrous cycle length after six months of observation (Table 1). Nevertheless, in individual females, estrus and diestrus phase prolongation and shortening of the proestrus phase, especially after six months was observed (Supplementary [Supplementary-material supplementary-material-1]).

### 3.4. Endometrial Epithelium Thickness

**Figure 6:**
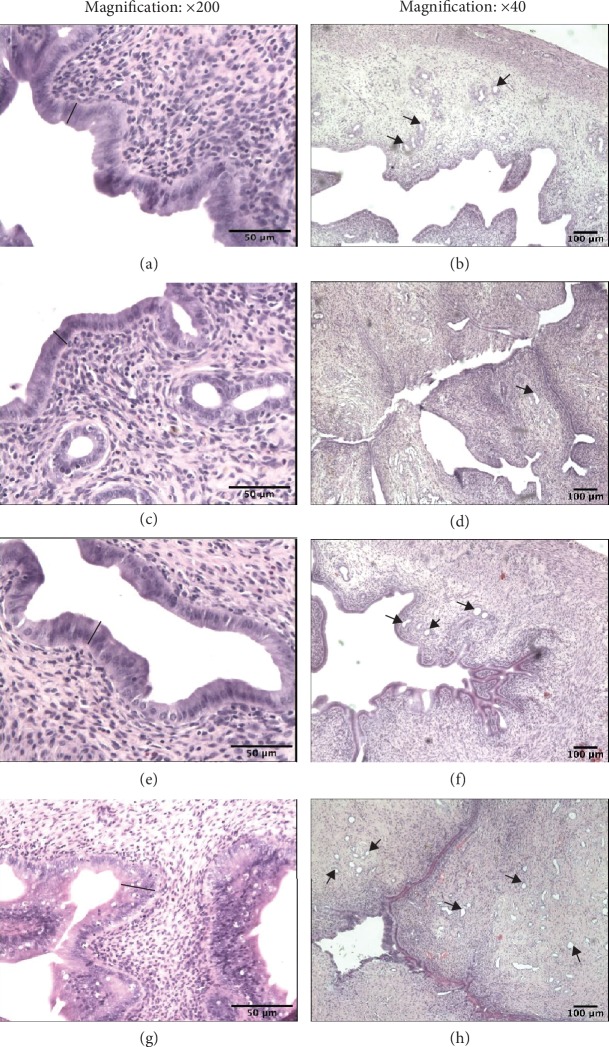
Effect of CdCl_2_ on uterine histology. Photomicrographs of uterine sections stained with hematoxylin and eosin from rats after a 180-day postexposure period (magnification: ×200 and ×40). (a and b) Sections from the uteri of pure control rats. A thick epithelial layer is seen as well as many glands. (c) Sections from the uteri of the Cd group (4.5 mgCd/kg). The epithelial layer is thin and contains a small number of the cells. (d) Sections from the uteri of the Cd group (4.5 mgCd/kg). The epithelial layer is thin, and the glands are not numerous. (e and f) Sections from the uteri of oil control rats. The epithelial layer is similar to that of the pure control rats. (g) Sections from the uteri of the 17*β*-estradiol group (0.03 mgE_2_/kg). Notice the increased thickness of the epithelial layer. (h) Sections from the uteri of the 17*β*-estradiol group (0.03 mgE_2_/kg). Numerous uterine glands are seen. Black bars represent epithelial thickness; arrows—uterine gland.

**Figure 7:**
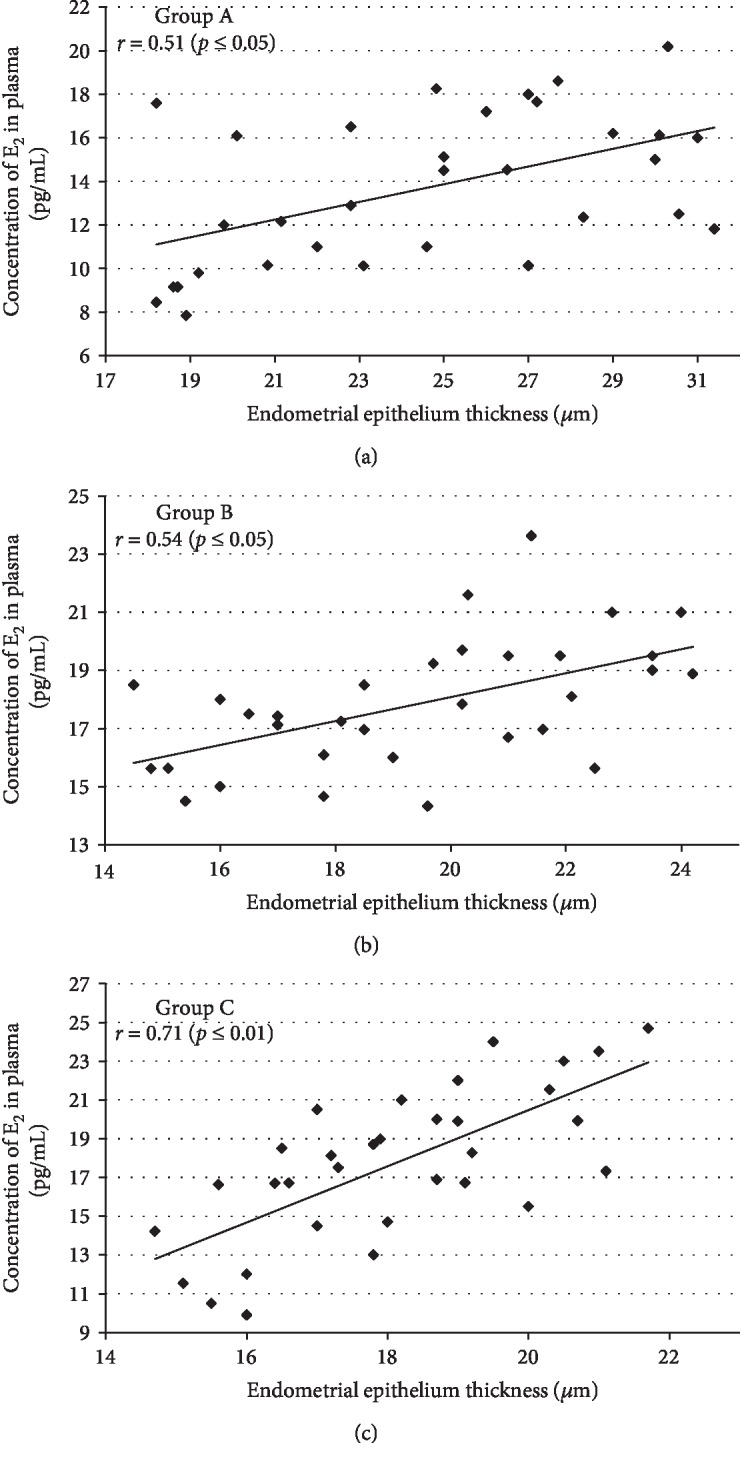
Association between plasma E_2_ concentration and endometrial epithelium thickness in Cd-exposed rats from group A (90-day exposure) (a); group B (90-day exposure and 90-day postexposure periods) (b); and group C (90-day exposure and 180-day postexposure periods) (c).

Morphometric analysis during the estrus stage showed that Cd administered orally (0.09, 0.9, 1.8, and 4.5 mg/kg b.w.) for 90 days induced a significantly increased thickness of the epithelial layer only after the smallest dose when compared to the pure control group (Table 2). Similar endometrium changes with increased glands in the uterus in the group of females treated with 17*β*-E_2_ were observed. These changes might suggest endometrial hyperplasia. Furthermore, the increase in endometrial epithelium thickness (1.6-fold) was detected in comparison to the oil and pure control groups, which persisted for six months following the termination of exposure to 17*β*-E_2_ (Table 2 and Figures 6(g) and (h)). In contrast, after the administration of higher doses of Cd, we observed a noticeable endometrium atrophy (a thin epithelial layer and not numerous glands), especially after a 180-day postexposure period, (Figures 6(c) and (d)) resulting from a decrease in E_2_, both in the plasma and in uterine tissue, confirmed by a significant correlation coefficient between the concentration of E_2_ in the plasma and endometrial epithelium thickness in all examined groups: *r* = 0.51 (*p* ≤ 0.05) for the exposure period (group A), *r* = 0.54 (*p* ≤ 0.05) after three months of observation (group B), and *r* = 0.71 (*p* ≤ 0.01) for the six-month observation period (group C) (Figure 7).

### 3.5. Ovary Histology

**Figure 8:**
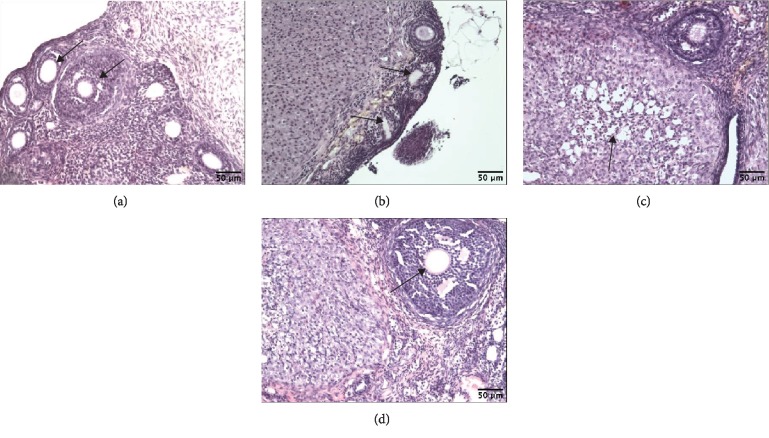
Effect of CdCl_2_ on ovary histology. Photomicrographs of ovary sections stained with hematoxylin and eosin (magnification: ×100) from rats after a 180-day postexposure period. (a) Sections from the ovaries of pure control rats. Notice numerous oocytes (arrows). (b) Sections from the ovaries of the Cd group (4.5 mg Cd/kg). The oocytes are scanty and two of them show degenerative changes (arrows). (c) Sections from the ovaries of the Cd group (4.5 mg Cd/kg). Arrow points—degenerative changes of corpus luteum. (d) Sections from the ovaries of the 17*β*-estradiol group (0.03 mg E_2_/kg). Corpus luteum and big follicle are seen (arrow—big follicle).

The control group demonstrated a normal basic structure of rat ovaries, usually containing a corpus luteum and all developmental stages of follicles (Figure 8(a)). The Cd-exposed group (4.5 mg/kg b.w.) showed histopathological alterations in the ovary: degeneration of the corpus luteum and damaged and less numerous oocytes (Figures 8(b) and 8(c)). In the positive control, no degenerative changes in the ovaries were reported (Figure 8(d)).

### 3.6. Oxidative Stress Parameters

Administration of Cd at the two highest doses (1.8 and 4.5 mg/kg b.w.) results in a significant rise of MDA concentration in the uterus (Table 3), but this effect persisted for the whole observation period following the exposure (up to six months) only after the highest dose (4.5 mg/kg b.w.). The same dose also affects the activity of the uterine CAT causing a significant decrease (around 25% compared to the pure control), but only just after the administration period. On the other hand, no changes in the concentration of GSH in this tissue were observed throughout all studied doses and periods. In the case of TAS, only the administration of E_2_ caused a remarkable increase of TAS in the plasma after the termination of exposure (Table 3). However, such effect was not noted in either observation periods or in the case of Cd exposure.

## 4. Discussion

There is a growing fear that a lifetime exposure to xenobiotics, which disturb hormonal homeostasis, may cause permanent change in the functioning of the organism, particularly in the reproductive system. Studies on the xenoestrogenicity of Cd conducted so far are ambiguous; some of them indicate its estrogenic activity [21, 25, 26, 40], while others show an antiestrogenic effect [21, 41, 42]. In the current study, to assess the estrogenic-like effect of Cd in female rats, we applied a 90-day Cd exposure model with a long observation period (up to six months) of exposed animals compared to control groups, including a positive control. We used *per os* administration, because this route is consistent with the general population's exposure to this metal. Average daily Cd intakes in European countries have been estimated between 0.00029 and 0.00106 mg/kg [4]. The lowest daily dose of Cd adopted in this study (0.09 mg/kg b.w.) was similar to a lower level of general population environmental exposure [43, 44]. In our study, Cd-B in rats exposed to 0.09 mg/kg b.w. for 90 days were similar to those observed in smokers in the general population [2]. Cd-B in long-term Cd exposure is thought to be a good indicator of its internal dose and accumulation, not only in the kidney (the target organ) but also in other soft tissues, including the uterus [45, 46]. The results obtained both in this study and in our previous studies (30-day administration of Cd to female rats) confirm the relationship between Cd-B and Cd uterus concentration [24]. After a 90-day administration of Cd, its concentration in the uterus increased in a dose-dependent manner and was about three times higher than that after a 30-day administration [24]. Moreover, Cd concentrations in the uterus six months after terminating the exposure were virtually at the same level as at the end of the administration period. Cd bioaccumulation in the uterine tissue was confirmed by numerous studies among smoking and nonsmoking women [11, 12, 46]. The health implications of Cd bioaccumulation in reproductive organs are still not well known.

Cd action as a metalloestrogen seems to depend not only on the level of exposure but also on its duration. While the administration of the lowest dose of Cd (0.09 mg/kg b.w.) caused estrogenic-like effects, in female rats which were administered higher doses, antiestrogenic effects were observed. The estrogenic effects of the lowest Cd dose were expressed by firstly, significantly higher E_2_ concentrations in the uterine tissue which correlated with the endometrial epithelium thickness, and secondly, by the prolongation of the estrus stage in the estrous cycle, like in positive control receiving 17*β*-E_2_. An abnormal cycle was maintained for up to six months after the termination of the administration.

Uterus weight is one of the accepted endpoints useful in estrogenicity assessment of xenobiotics [47, 48]. Although changes in the endocrine profile or thickness of the epithelial endometrium may affect the weight of the uterus, in our study, the wet weight of the uterus was not significantly different compared to the pure control. Ali et al. [27] did not detect any changes in the wet weight of the uterus either, but they did notice an increase in the uterine luminal epithelium in mice receiving Cd in doses of 50 and 500 *μ*g/kg b.w. Similarly, no changes in uterine weight due to Cd have been described in other studies [21, 49]; however, Johnson et al. [26] showed a significant increase in uterine weight in rats after a single *i.p.* injection of Cd at 5 *μ*g/kg b.w.

The mechanism of the estrogenic action of Cd in the uterus is thought to be associated with the ER. Stoica et al. [25] suggest that Cd ions can activate the ER by creating high-affinity interactions with the hormone receptor binding domain. Fechner et al. [50] revealed that Cd interacts with the ligand binding domain (LBD) of the ER*α* and affects the conformation of the receptor. However, the binding event, as well as the induced conformation change, greatly depends on the accessibility of the cysteine tails in the LBD. Kluxen et al. [51] showed that Cd exerts estrogen-like effects and modulates aryl hydrocarbon receptor (AhR) expression and that of AhR target genes. There are also studies indicating that Cd does not activate classical estrogen signaling in the uterus but interferes with its pathway [27].

The fact that the estrogen response induced by Cd is dose-dependent seems to be confirmed by *in vitro* studies. For instance, Denier et al. [52] demonstrated on yeast cells that Cd in low concentrations enhanced the estrogenic response due to the interaction of E_2_ with ER. Importantly, in high concentrations, Cd was cytotoxic to yeast cells. The dose-dependent effect of Cd was also observed in our study. The administration of Cd at high doses (0.9-4.5 mg/kg b.w.) to female rats led to a decrease in E_2_ concentration in both plasma and uterine tissue. Although the effect of lowering E_2_ concentrations in plasma by Cd exposure has been previously described [18, 19, 22], the same effect in the uterine tissue is, according to our knowledge, described here for the first time.

Disorders in E_2_ secretion may have various causes, including, for example, ovary damage, which was observed in this study and was expressed as, among others, degeneration of the corpus luteum, damaged and less numerous oocytes, and degeneration of the granulosa cells. These effects are in agreement with the literature [20, 28–30]. Structural damage to the ovary and disturbed E_2_ secretion noted after the highest Cd dose may contribute to the prolongation of the diestrus stage in the estrous cycle. Such observations are in line with our previous results from a 30-day experiment [24] and studies conducted by Samuel et al. [29]. Disorders in the cycle may be the first symptoms of reproductive aging [53]. This can be indirectly confirmed by decreased thickness of the epithelial endometrium observed in our study, which significantly correlated with a lowered concentration of E_2_ in the plasma.

It is suggested that estradiol is a physiological antioxidant whose deficiency has been shown to be associated with oxidative stress [54]. Thus, the changes observed in the uterus of rats receiving high doses of Cd in this study may also be associated with the induction of oxidative stress in the reproductive organs and it cannot be ruled out that the induction of oxidative stress contributes to changes in hormonal balance. The mechanism of prooxidative Cd action is well known [55]. Although Cd is not a redox-active agent and is not able to directly generate ROS through the Fenton or Haber-Weiss reaction, it contributes to the occurrence of oxidative stress indirectly through weakening the antioxidative defence and increasing prooxidant concentrations, as well as causing mitochondria injury [56]. Numerous *in vitro* and *in vivo* studies confirmed that Cd disrupts the antioxidant defence by reducing the levels of antioxidants, which are important in the removal of ROS [55, 57]. Effects of ROS can be mitigated by antioxidants, both nonenzymatic and enzymatic, and key enzymatic antioxidants in cells are superoxide dismutase (SOD), catalase (CAT), and glutathione peroxidase (GPx) [58, 59]. CAT plays a crucial role in scavenging peroxides such as H_2_O_2_. Thus, its diminished activity may indicate cellular accumulation of this reactive oxygen species, which are capable of crossing cell membranes [58]. Although subacute (up to 30 days) administration of Cd leads to alterations in enzymatic antioxidant defence in rat uteri and ovaries [29, 31, 60–62], there is no data concerning subchronic Cd exposure. In the present subchronic study, decreased CAT activity and increased MDA concentration caused by the highest Cd dose seem to confirm disturbed redox balance in the uterus. Although other markers of oxidative stress were not determined, it could be assumed that the decrease of CAT activity may indicate an oxidative stress in this tissue.

It was reported that ROS may propagate the initial attack on lipid membranes to cause lipid peroxidation (LPO). MDA (malondialdehyde) is one of the numerous compounds formed during the process of LPO, which binds to, inter alia, proteins and DNA modifying its structure. Studies proved that these MDA adducts play a critical role in many cellular processes and may participate in secondary deleterious reactions that may cause profound changes in the biochemical properties of biomolecules, which may facilitate development of various pathological states [63]. The significantly increased MDA concentration in uterine tissue, which was observed in this study, remained up to six months after terminating the exposure. Thus, the histopathological changes in the uterus may be also the result of the indirect induction of lipid peroxidation by Cd.

Noteworthy, Cd could induce ROS production also nonenzymatically. Nonenzymatic antioxidants such as vitamins C and E, zinc and selenium, protein thiols (e.g., glutathione), bilirubin, and urate are often determined as total antioxidant status (TAS). In this study, the uterine concentration of GSH and plasma TAS in rats exposed to Cd did not differ significantly from that of control rats. Also, Mężyńska et al. [44, 64] did not observe a statistically significant difference in TAS concentrations analyzed in rat liver after a similar time (3 month) and level of Cd exposure (1 and 5 mg/kg b.w.). A significantly reduced hepatic TAS concentration was noticed only after ten months of Cd administration [44, 64]. Therefore, it has been suggested that the mechanism of oxidative stress induced by Cd may vary and depend on the time of exposure. Some studies indicate that chronic exposure to Cd probably induces ROS by overwhelming the antioxidant defence leading to increased lipid peroxidation [64, 65]. On the other hand, short-term exposure to Cd contributes to a significant depletion of glutathione and protein-bound sulfhydryl groups, which results in enhanced production of ROS [66, 67].

Unfortunately, it is very difficult to state whether Cd can affect reproduction in women through changes in sex hormones and/or through oxidative stress. So far, it has only been shown that Cd accumulates in women's uteri and it may also be responsible for impaired hormonal metabolism [10, 68–72]. Moreover, in several epidemiological studies, the relationship between dietary Cd exposure and incidence of hormone-related cancer, including in the endometrium and ovaries, has been observed [11, 73].

## 5. Conclusions

Our study indicates that Cd exhibits both estrogenic-like (in a low dose range) and antiestrogenic-like (in a high dose range) effects, which were connected with disturbed plasma and the uterine estradiol concentrations and estrous cyclicity disorders. These effects, as well as increased lipid peroxidation in the uterus and ovary, were observed for six months following termination of Cd exposure. In addition, Cd toxicity after the highest dose was also associated with structural damage of the uterus and ovaries. The obtained results indicate that women's long-term exposure to Cd may lead to reproductive system disorders, which might even result in infertility.

## Figures and Tables

**Table 1:** Cycle phases and all cycle lengths following oral, subchronic CdCl_2_, or 17*β*-estradiol (positive control) exposure and 90- and 180-day postexposure periods in comparison to controls.

Treatment	Dose (mg/kg b.w.)	Phase length (days)				Cycle length (days)	No. of extended cycles
		Proestrus	Estrus	Metaestrus	Diestrus		
Group A (90-day exposure)							
Pure control	0	1.8 ± 0.1	2.8 ± 0.2	2.6 ± 0.1	4.7 ± 0.2	4.3 ± 0.1	0
Cd	0.09	1.2±0.1^a^	4.6 ± 1.0^a^	1.9 ± 0.3^a^	4.6 ± 0.7	5.4 ± 0.8^a^	4
Cd	0.9	1.8 ± 0.2	2.9 ± 0.2	2.2 ± 0.2	5.1 ± 0.2^a^	4.7 ± 0.2^a^	3
Cd	1.8	1.8 ± 0.2	3.4 ± 0.2^a^	2.3 ± 0.2	4.4 ± 0.2	4.9 ± 0.3^a^	3
Cd	4.5	1.4 ± 0.1^a^	2.8 ± 0.2	2.6 ± 0.1	5.2 ± 0.2^a^	4.5 ± 0.3	4
Oil control	0	2.3 ± 0.1	2.6 ± 0.1	2.9 ± 0.2	4.3 ± 0.2	3.9 ± 0.2	0
Positive control	0.03	1.4 ± 0.1^a^	4.6 ± 0.4^a^	2.3 ± 0.2	3.7 ± 0.3^a^	5.6 ± 0.3^a^	5

Group B (90-day exposure and 90-day postexposure periods)							
Pure control	0	1.7 ± 0.1	2.3 ± 0.1	2.8 ± 0.2	4.3 ± 0.2	3.8 ± 0.1	0
Cd	0.09	1.7 ± 0.1	1.9 ± 0.3^a^	2.6 ± 0.3	4.8 ± 0.4	4.8 ± 0.5^a^	2
Cd	0.9	1.6 ± 0.1	2.9 ± 0.2^a^	2.2 ± 0.2^a^	4.0 ± 0.2	4.1 ± 0.1	0
Cd	1.8	1.6 ± 0.1	2.6 ± 0.2	2.6 ± 0.1	4.1 ± 0.1	4.0 ± 0.1	0
Cd	4.5	1.8 ± 0.2	2.4 ± 0.2	2.8 ± 0.2	4.0 ± 0.2	3.9 ± 0.1	0
Oil control	0	1.6 ± 0.1	2.3 ± 0.2	2.9 ± 0.2	4.1 ± 0.2	4.0 ± 0.1	0
Positive control	0.03	1.6 ± 0.1	2.2 ± 0.1	2.7 ± 0.2	4.4 ± 0.1	4.1 ± 0.1	0

Group C (90-day exposure and 180-day postexposure periods)							
Pure control	0	1.6 ± 0.1	2.8 ± 0.1	3.1 ± 0.3	4.4 ± 0.1	4.1 ± 0.1	0
Cd	0.09	1.5 ± 0.3	4.9 ± 0.9^a^	2.7 ± 0.3	2.9 ± 0.4^a^	5.3 ± 0.9^a^	3
Cd	0.9	1.6 ± 0.2	4.1 ± 0.6^a^	2.8 ± 0.2	3.5 ± 0.3^a^	4.3 ± 0.3	1
Cd	1.8	1.6 ± 0.1	2.8 ± 0.1	3.4 ± 0.1	4.2 ± 0.1	4.0 ± 0.1	0
Cd	4.5	1.8 ± 0.1	2.8 ± 0.1	3.6 ± 0.3	3.9 ± 0.3	4.2 ± 0.1	0
Oil control	0	1.4 ± 0.1	2.8 ± 0.1	3.2 ± 0.2	4.7±0.2	4.1 ± 0.1	0
Positive control	0.03	1.4 ± 0.1	2.9 ± 0.1	3.3 ± 0.2	4.1 ± 0.2	4.3 ± 0.2	1

All values are expressed as means ± SEM (*n* = 8). ^a^Significantly different from pure control (*p* ≤ 0.05).

**Table 2:** Endometrial epithelium thickness (*μ*m) after subchronic oral exposure to CdCl_2_ or 17*β*-estradiol (E_2_) exposure and 90- and 180-day postexposure periods in comparison to controls.

Groups	Endometrial epithelium thickness (*μ*m)						
	Pure control	0.09 mgCd/kg	0.9 mgCd/kg	1.8 mgCd/kg	4.5 mgCd/kg	Oil control	Positive control
A—90-day exposure	19.2 ± 2.2	27.0 ± 3.1^a,c^	21.7 ± 3.6^c^	20.1 ± 3.1^c^	24.5 ± 6.3^c^	21.9 ± 3.3	36.4 ± 2.5^b^
B—90-day postexposure period	21.8 ± 4.3	21.9 ± 1.9^c^	19.4 ± 2.1^c^	17.9 ± 2.1^c^	19.1 ± 2.9^c^	21.80 ± 1.7	34.8 ± 2.5^b^
C—180-day postexposure period	21.7 ± 1.7	19.6 ± 1.3^c^	18.9 ± 2.5^c^	17.1 ± 1.7^a,b,c^	16.5 ± 1.6^a,b,c^	22.5 ± 2.1	33.8 ± 4.1^b^

All values are expressed as mean ± SD. ^a^*p* ≤ 0.05, significantly different from pure control animals. ^b^*p* ≤ 0.0, significantly different from oil control. ^c^*p* ≤ 0.05, significantly different from positive control.

**Table 3:** Oxidative stress biomarkers in rats after oral exposure to CdCl_2_ or 17*β*-estradiol (positive control) exposure and 90- and 180-day postexposure periods in comparison to controls.

Treatment	Doses (mg/kg b.w.)	TAS in plasma (mM)	GSH in uterus (*μ*mol/g)	CAT in uterus (U/mg protein)	MDA in uterus (nmol/g)
Group A (90-day exposure)					
Pure control	0	1.16 ± 0.09	1.37 ± 0.32	8.66 ± 0.96	62.3 ± 6.80
Cd	0.09	1.11 ± 0.09	1.47 ± 0.46	11.0 ± 2.10	80.2 ± 7.05
Cd	0.9	1.08 ± 0.04	1.84 ± 0.18	12.3 ± 1.54	71.0 ± 14.2
Cd	1.8	1.02 ± 0.07	1.20 ± 0.41	8.71 ± 0.89	84.7 ± 4.25^a^
Cd	4.5	0.88 ± 0.15	1.53 ± 0.40	6.61 ± 0.60^a,b,c^	98.1 ± 5.13^a,b,c^
Oil control	0	0.95 ± 0.11	1.80 ± 0.10	8.99 ± 1.68	61.8 ± 3.52
Positive control	0.03	1.61 ± 0.60^b^	1.56 ± 0.33	7.99 ± 1.53	70.0 ± 8.52

Group B (90-day exposure and 90-day postexposure periods)					
Pure control	0	1.04 ± 0.09	1.46 ± 0.31	9.02 ± 1.60	65.2 ± 11.30
Cd	0.09	1.01 ± 0.06	1.24 ± 0.21	9.88 ± 1.82	74.5 ± 10.92
Cd	0.9	1.00 ± 0.03	1.14 ± 0.19	11.0 ± 1.54	74.8 ± 14.64
Cd	1.8	0.96 ± 0.07	1.32 ± 0.20	7.89 ± 1.15	72.9 ± 9.07
Cd	4.5	0.93 ± 0.08	1.29 ± 0.05	7.25 ± 1.61	94.6 ± 19.50^a^
Oil control	0	1.05 ± 0.06	1.41 ± 0.12	9.56 ± 1.98	67.0 ± 5.04
Positive control	0.03	1.18 ± 0.06	1.61 ± 0.54	10.2 ± 1.63	68.9 ± 9.02

Group C (90-day exposure and 180-day postexposure periods)					
Pure control	0	1.02 ± 0.08	1.14 ± 0.24	9.66 ± 1.36	60.5 ± 5.85
Cd	0.09	0.95 ± 0.07	1.25 ± 0.29	9.98 ± 1.95	58.2 ± 13.90
Cd	0.9	0.98 ± 0.04	1.08 ± 0.12	10.6 ± 1.83	71.6 ± 11.08
Cd	1.8	0.96 ± 0.09	0.99 ± 0.08	9.21 ± 1.39	75.3 ± 5.33
Cd	4.5	0.95 ± 0.10	1.25 ± 0.49	8.98 ± 1.65	92.5 ± 7.10^a,b,c^
Oil control	0	1.02 ± 0.06	1.21 ± 0.24	10.0 ± 0.91	64.9 ± 6.93
Positive control	0.03	1.16 ± 0.09	1.15 ± 0.25	11.1 ± 1.66	62.7 ± 3.96

All values are expressed as mean ± SD. ^a^*p* ≤ 0.05, significantly different from control pure animals. ^b^*p* ≤ 0.05, significantly different from oil control, ^c^*p* ≤ 0.05, significantly different from positive control.

## Data Availability

The data used to support the findings of this study are included within the article.

## Supplementary Materials

Supplementary Materials The supplemental information provides additional data concerning organs and body weights of rats after oral exposure to CdCl_2_ or 17*β*-estradiol (positive control) and 90 and 180-day postexposure periods in comparison to controls (Tables S1 and S2). Moreover, Figures S1, S2, and S3 depict the estrous cycle in single females after all periods of exposure to Cd or 17*β*-estradiol as well as in observation periods.
