# Supplementary material for: Subchronic Exposure to Cadmium Causes Persistent Changes in the Reproductive System in Female Wistar Rats
Source: Oxid Med Cell Longev. 2019 Dec 17;2019:6490820. doi: 10.1155/2019/6490820 (PMC6935823; doi:10.1155/2019/6490820)
Supplement: Supplementary Materials — The supplemental information provides additional data concerning organs and body weights of rats after oral exposure to CdCl2 or 17β-estradiol (positive control) and 90 and 180-day postexposure periods in comparison to controls (Tables S1 and S2). Moreover, Figures S1, S2, and S3 depict the estrous cycle in single females after all periods of exposure to Cd or 17β-estradiol as well as in observation periods. [file 6490820.f1.docx]

*SUPPLEMENTARY MATERIALS*

Subchronic exposure to cadmium causes persistent changes in the reproductive system in female Wistar rats

**Marzenna Nasiadek^1^*, Marian Danilewicz^2^, Michał Klimczak^1^, Joanna Stragierowicz^1^, Anna Kilanowicz^1^**

^1^Department of Toxicology, Medical University of Lodz, Muszynskiego 1, 90-151 Lodz, Poland

^2^ Department of Pathology, Medical University of Lodz, Pomorska 251, 92-213 Lodz, Poland

***Corresponding author:** Marzenna Nasiadek,

e-mail address: [marzenna.nasiadek@umed.lodz.pl](mailto:marzenna.nasiadek@umed.lodz.pl)

Supplemental information:

The supplemental information provide additional data concerning organs and body weights of rats after oral exposure to CdCl_2_ or 17β-estradiol (positive control) and 90, 180-day post-exposure period in comparison to controls (Tables S1 and S2). Moreover, the figures (S1, S2 and S3) depict the estrous cycle in single females after all periods of exposure to Cd or 17β-estradiol as well as in observation periods.

Table S1: Initial and final body weight of rats after oral exposure to CdCl_2_ or 17β-estradiol (positive control) and 90-, 180-day post-exposure
 period in comparison to controls

| **Treatment** | **Doses**  **[mg/kg b.w.]** |  | **Body weight** |  |  |
| --- | --- | --- | --- | --- | --- |
|  |  | **At the beginning**  **[g]** | **At the end**  **[g]** | **Change** | |
|  |  |  |  | **[g]** | **[% of initial**  **body weight]** |
| **Group A (90-day exposure)** | | | | | |
| **Pure control** | 0 | 207 ± 5.5 | 237 ± 11.9 | 29.8 ± 7.4 | 14.3 |
| **Cd** | 0.09 | 211 ± 5.6 | 234 ± 12,4 | 22.8 ± 8.8 | 10.7 |
| **Cd** | 0.9 | 210 ± 6.0 | 236 ± 10.8 | 26.3 ± 8.5 | 12.5 |
| **Cd** | 1.8 | 210 ± 5.5 | 239 ± 9.7 | 29.4 ± 8.0 | 14.0 |
| **Cd** | 4.5 | 210 ± 5.3 | 238 ± 12.5 | 28.4 ± 9.3 | 13.5 |
| **Oil control** | 0 | 205 ± 5.7 | 236 ± 11.8 | 31.3 ± 9.0 | 15.3 |
| **Positive control** | 0.03 | 203 ± 5.2 | 230 ± 9.6 | 26.1 ± 8.7 | 12.9 |
| **Group B (90-day exposure and 90-day post-exposure period)** | | | | | |
| **Pure control** | 0 | 204 ± 5.4 | 265 ± 13.8 | 61.1 ± 14.2 | 30.0 |
| **Cd** | 0.09 | 205 ± 5.7 | 268 ± 14.1 | 63.6 ± 10.9 | 31.0 |
| **Cd** | 0.9 | 203 ± 5.6 | 260 ± 14.3 | 57.2 ± 14.6 | 28.3 |
| **Cd** | 1.8 | 206 ± 6.8 | 267 ± 13.1 | 61.4 ± 13.9 | 30.0 |
| **Cd** | 4.5 | 204 ± 5.3 | 263 ± 16.2 | 58.8 ± 16.0 | 28.8 |
| **Oil control** | 0 | 207 ± 5.5 | 261 ± 18.7 | 54.1 ± 14.5 | 26.1 |
| **Positive control** | 0.03 | 205 ± 6.6 | 261 ± 14.3 | 56.1 ± 13.4 | 27.4 |
| **Group C (90-day exposure and 180-day post-exposure period)** | | | | | |
| **Pure control** | 0 | 200 ± 5.2 | 252 ± 17.3 | 52.2 ± 15.9 | 26.1 |
| **Cd** | 0.09 | 208 ± 7.3 | 260 ± 10.3 | 51.0 ± 12.8 | 24.6 |
| **Cd** | 0.9 | 205 ± 5.5 | 251 ± 17.1 | 45.3 ± 19.4 | 22.2 |
| **Cd** | 1.8 | 207 ± 5.8 | 262 ± 18.8 | 54.6 ± 19.6 | 26.4 |
| **Cd** | 4.5 | 206 ± 6.8 | 250 ± 14.3 | 43.6 ± 18.1 | 21.4 |
| **Oil control** | 0 | 204 ± 6.0 | 273 ± 18.0 | 68.8 ± 18.9 | 33.7 |
| **Positive control** | 0.03 | 206 ± 5.2 | 256 ± 19.0 | 50.0 ± 19.3 | 24.4 |

All values are expressed as mean ± SD

Table S2: Liver, kidney and uterine weight of rats after oral exposure to CdCl_2_ or 17β-estradiol (positive control) and 90-, 180-day post-exposure period in comparison to controls

| **Treatment** | **Dose [mg/kg b.w.]** | **Liver** | | **Kidneys** | | **Uterus** | |
| --- | --- | --- | --- | --- | --- | --- | --- |
|  |  | **weight**  **[g]** | **% of final body weight**  **[g/100 g b.w.]** | **weight**  **[g]** | **% of final body weight**  **[g/100 g b.w.]** | **weight**  **[g]** | **% of final body weight**  **[g·100 g b.w.^-1^]** |
| **Group A (90-day exposure)** | | | | | | | |
| **Pure control** | 0 | 6.89 ± 0.76 | 2.91 ± 0.31 | 1.21 ± 0.12 | 0.51 ± 0.05 | 0.88 ± 0.19 | 0.37 ± 0.08 |
| **Cd** | 0.09 | 6.64 ± 0.59 | 2.85 ± 0.33 | 1.17 ± 0.10 | 0.50 ± 0.05 | 0.95 ± 0.15 | 0.41 ± 0.07 |
| **Cd** | 0.9 | 6.94 ± 0.51 | 2.95 ± 0.25 | 1.31 ± 0.13 | 0.56 ± 0.07 | 0.92 ± 0.15 | 0.39 ± 0.06 |
| **Cd** | 1.8 | 7.47 ± 0.79 | 3.12 ± 0.33 | 1.40 ± 0.10 | 0.59 ± 0.04 | 0.82 ± 0.14 | 0.34 ± 0.06 |
| **Cd** | 4.5 | 6.91 ± 0.74 | 2.90 ± 0.21 | 1.32 ± 0.12 | 0.55 ± 0.04 | 0.90 ± 0.14 | 0.38 ± 0.06 |
| **Oil control** | 0 | 6.58 ± 0.65 | 2.79 ± 0.26 | 1.28 ± 0.15 | 0.54 ± 0.07 | 0.87 ± 0.11 | 0.37 ± 0.05 |
| **Positive control** | 0.03 | 6.97 ± 0.54 | 3.04 ± 0.25 | 1.23 ± 0.12 | 0.54 ± 0.06 | 0.85 ± 0.09 | 0.37 ± 0.04 |
| **Group B (90-day exposure and 90-day post-exposure period)** | | | | | | | |
| **Pure control** | 0 | 7.84 ± 0.50 | 2.96 ± 0.22 | 1.32 ± 0.10 | 0.50 ± 0.04 | 1.07 ± 0.19 | 0.40 ± 0.08 |
| **Cd** | 0.09 | 8.17 ± 0.57 | 3.05 ± 0.23 | 1.37 ± 0.09 | 0.51 ± 0.03 | 0.99 ± 0.16 | 0.37 ± 0.06 |
| **Cd** | 0.9 | 7.69 ± 1.16 | 2.97 ± 0.46 | 1.28 ± 0.18 | 0.49 ± 0.07 | 1.09 ± 0.37 | 0.42 ± 0.15 |
| **Cd** | 1.8 | 8.00 ± 0.44 | 3.00 ± 0.22 | 1.40 ± 0.09 | 0.53 ± 0.05 | 1.01 ± 0.18 | 0.38 ± 0.07 |
| **Cd** | 4.5 | 7.81 ± 1.12 | 2.97 ± 0.38 | 1.32 ± 0.15 | 0.50 ± 0.05 | 0.92 ± 0.18 | 0.35 ± 0.07 |
| **Oil control** | 0 | 7.37 ± 0.61 | 2.83 ± 0.31 | 1.45 ± 0.10 | 0.56 ± 0.05 | 0.95 ± 0.15 | 0.33 ± 0.07 |
| **Positive control** | 0.03 | 8.38 ± 0.84 | 3.22 ± 0.39 | 1.51 ± 0.13 | 0.58 ± 0.07 | 0.95 ± 0.09 | 0.37 ± 0.03 |
| **Group C (90-day exposure and 180-day post-exposure period)** | | | | | | | |
| **Pure control** | 0 | 7.45 ± 0.68 | 2.95 ± 0.15 | 1.23 ± 0.14 | 0.49 ± 0.04 | 1.12 ± 0.31 | 0.45 ± 0.13 |
| **Cd** | 0.09 | 7.41 ± 0.61 | 2.87 ± 0.31 | 1.31 ± 0.11 | 0.50 ± 0.05 | 1.14 ± 0.37 | 0.38 ± 0.19 |
| **Cd** | 0.9 | 7.71 ± 0.47 | 3.09 ± 0.27 | 1.38 ± 0.10 | 0.55 ± 0.03 | 1.32 ± 0.29 | 0.53 ± 0.12 |
| **Cd** | 1.8 | 8.32 ± 0.46 | 3.19 ± 0.19 | 1.46 ± 0.13 | 0.56 ± 0.03 | 1.36 ± 0.23 | 0.52 ± 0.08 |
| **Cd** | 4.5 | 7.71 ± 0.71 | 3.09 ± 0.31 | 1.46 ± 0.12 | 0.58 ± 0.05 | 1.31 ± 0.31 | 0.52 ± 0.10 |
| **Oil control** | 0 | 7.58 ± 0.51 | 2.79 ± 0.28 | 1.40 ± 0.07 | 0.51 ± 0.04 | 1.22 ± 0.21 | 0.45 ± 0.09 |
| **Positive control** | 0.03 | 7.26 ± 0.66 | 2.85 ± 0.29 | 1.28 ± 0.13 | 0.50 ± 0.03 | 1.09 ± 0.18 | 0.43 ± 0.08 |

All values are expressed as mean ± SD

B

A

C

D

F

E

G

Figure S1: Depiction of estrous cycle after 90 days treatment. Each point (different number) depicts the cycle phase: 1 (estrus); 1.5 (cycle phase between estrus and metaestrus); 2 (metaestrus); 2.5 (cycle phase between metaestrus and diestrus), 3 (diestrus); 3.5 (cycle phase between diestrus and proestrus); 4 (proestrus); 4.5 (cycle phase between proestrus and estrus). Vertical lines separate individual rats. (A) pure control; (B) Cd 0.09 mg/kg b.w.; (C) Cd 0.9 mg/kg b.w.; (D) Cd 1.8 mg/kg b.w.; (E) Cd 4.5 mg/kg b.w.; (F) oil control; (G) positive control

B

A

D

C

E

F

G

Figure S2: Depiction of estrous cycle after 90 days treatment and 90-day post-exposure period. Each point (different number) depicts the cycle phase: 1 (estrus); 1.5 (cycle phase between estrus and metaestrus); 2 (metaestrus); 2.5 (cycle phase between metaestrus and diestrus), 3 (diestrus); 3.5 (cycle phase between diestrus and proestrus); 4 (proestrus); 4.5 (cycle phase between proestrus and estrus). Vertical lines separate individual rats. (A) pure control; (B) Cd 0.09 mg/kg b.w.; (C) Cd 0.9 mg/kg b.w.; (D) Cd 1.8 mg/kg b.w.; (E) Cd 4.5 mg/kg b.w.; (F) oil control; (G) positive control

A

B

C

D

E

F

G

Figure S3: Depiction of estrous cycle after 90 days treatment and 180-day post-exposure period. Each point (different number) depicts the cycle phase: 1 (estrus); 1.5 (cycle phase between estrus and metaestrus); 2 (metaestrus); 2.5 (cycle phase between metaestrus and diestrus), 3 (diestrus); 3.5 (cycle phase between diestrus and proestrus); 4 (proestrus); 4.5 (cycle phase between proestrus and estrus). Vertical lines separate individual rats. (A) pure control; (B) Cd 0.09 mg/kg b.w.; (C) Cd 0.9 mg/kg b.w.; (D) Cd 1.8 mg/kg b.w.; (E) Cd 4.5 mg/kg b.w.; (F) oil control; (G) positive control
